# Simple, Yet Effective Means for Making Attachment to Teeth with Vital Pulps, for Removable Bridges and Partial Plates*Read before the National Dental Association at its Twenty-first Annual Session, New York City, N. Y., October 23-26, 1917, and reprinted from July, 1918, *Journal N. D. A*.

**Published:** 1918-12

**Authors:** G. Walter Dittmar


### SIMPLE, YET EFFECTIVE MEANS FOR MAKING ATTACHMENT TO TEETH WITH VITAL PULPS, FOR REMOVABLE BRIDGES AND PARTIAL PLATES *

BY G. WALTER DITTMAR, D.D.S.

In the limited time allotted me, it will be impossible to give attention to other than principles and go briefly into the technic of construction of but two forms of tenso-friction appliances used as attachments to teeth with vital pulps.

In the various text-books on prosthetic dentistry and in the numerous dental journals all of the imaginable forms of attachment appliances, operating by friction produced by tension, have been described and more or less definitely illustrated.

Perhaps the most comprehensive expose of the subject is that which was presented to the Illinois State Dental Society in 1915, and also in 1917, by Dr. Chas. W. Coltrin, of Chicago. These articles are published in the proceedings of the Illinois State Dental Society and also in the *Dental Review*, October, 1915, and September, 1917, and are worthy of careful reading, for they described prac-

---

* Read before the National Dental Association at its Twenty-first Annual Session, New York City, N. Y., October 23-26, 1917, and reprinted from July, 1918, *Journal N. D. A.*

tically all of the patented forms of attachments and the various forms of clasps used by dentists.

Before considering the mechanical phase of the two forms of attachments alluded to, permit me to call attention to at least three fundamental questions that every prosthetist should have in mind when a case presents requiring some form of attachment.

The first is—How can the tenso-friction appliance be constructed so that it and the partial denture will do the least possible harm to the peridental membrane and other supporting tissues of the tooth, to which anchorage is made?

The second is—How can it be constructed that it will produce the least possible harm to the enamel and other hard tooth tissues through wear and promotion of conditions which may produce caries?

The third is—How can the attachment be constructed so it will securely hold the partial denture in position?

The latter unfortunately too often seems to be the first and only thought the dentist has in mind; apparently giving no consideration to the serious consequences which so often follow where the wrong type of attachment is used viz., the producing of disease of the hard tissues or of the supporting tissues of the teeth, or both.

I well realize that it is practically impossible to attach to any tooth with the very best form of attachment without some harm resulting to that tooth ultimately, because of the attachment.

The point is—*Use the type or form of attachment that will produce the least possible harm and still give sufficient retention to the partial artificial denture.*

The crying demand of our profession as well as of many of our patients is that more so-called removable bridges and partial plates be made and less fixed bridges. Also that we shall make attachment to vital teeth, if the teeth to which attachment must be made are vital and healthy. May I add that as a rule, attachment to pulpless teeth is a comparatively simple procedure, though good judgment and fine skill are demanded to obtain the best results.

In making attachment to vital teeth two methods present themselves: First —the use of some of the patented forms of attachments like the Roach, Gilmore, Morgan, etc., soldered to an inlay or crown, necessitating the mutilation of the tooth.

Second—the attachment made by some form of clasp without mutilation of the tooth.

I will consider first that method which requires mutilation of the tooth to which anchorage is made. A well-made, securely-anchored inlay is usually sufficient to hold the attachment for a removable partial denture and where conditions require it, a mesio-occluso-distal inlay, and onlay, a pinlay, or a lingual-hood crown provide most stable anchorage.

Confining my remarks to removable partial dentures, and more in particular to that class where all the posterior teeth are missing and all of the anterior teeth are in position, conditions very frequently found in the lower as well as in the upper, I would select as the best form, the well known Roach attachment.

I would use this attachment because *it produces the least possible strain upon the anchor tooth* or, to be more explicit, the tooth to which anchorage is made.

There are two forms of this attachment, both operating on the same principle, viz., a ball or button attached to the anchor tooth and a clasp or tube fastened to the removable denture.

The button of the Roach attachment should be soldered to the inlay or crown about one thirty-second of an inch from the gum and it is advisable to set it as far lingually as possible, but have it project distally parallel with the alveolar ridge. Not lingually to interfere with the tongue.

The tube must be modified by soldering to it a clasp metal wire or strip of plate clasp metal so bent that one end will act as an anchorage lug which is to be soldered or vulcanized to the removable denture, the other end to act as a contact spur, resting on the proximal surface of the inlay or crown to which anchorage is made. The function of this contact spur is to produce "indirect retention." The tube gripping the button near the gingival portion of the tooth and the contact spur bent to make tight contact near the occlusal or incisal portion of the proximal surface of the tooth will cause the saddle or base to lie in contact with the mucous membrane.

If plate clasp metal is used for making the contact spur use not less than twenty-six (26) gauge. Cut a piece about as wide as the diameter of the tube and long enough when bent to fit along the back and over the end of the tube; again bending the part which lays over the end almost at right angles away from the tube, cutting it off even with the occlusal or incisal portion of the tooth when the finished clasp is in position.

The other end of the strip have long enough and bent at an angle to reach the metal saddle or extend as an anchorage lug into the vulcanite.

Now slightly flatten the back of the tube (opposite the open part), with a stone or file. Place anti-flux so the part of the strip which is to fit over the end of the tube will not solder. Then solder the flattened part of the tube to the strip using a minimum of 18 karat gold solder. If a 16 gauge clasp metal or elastic wire is used, slightly flatten the tube on one side, also the wire. Solder the wire to the tube, slightly flatten the end of wire and bend to form a contact spur, the other end of the wire to be formed so it will act as an anchorage lug which can be soldered to a metal saddle or attached to the vulcanite.

The tube of the new form of the Roach attachment is so made by the manufacturer that it has the modifications just described, viz., the tube, contact spur and anchorage lug are all made from a single piece of clasp metal. This makes it more compact and decidedly more simple and convenient in its application.

The button of the new form is oval, somewhat like an old-fashioned door knob—and is made with a wide flanged flat base instead of the constricted projection which is to be soldered to the inlay or crown. This in many cases makes it more convenient for attachment with solder and also because of the comparatively large base makes a more secure union when soldered.

The same rules which apply in the use of the older form must be observed in the application and use of the new form, as regards the position of attachment to the inlay or crown. The new form, however, affords another very excellent method of using it in those cases where anchorage to a saddle is made at one end only: viz., solder a strip of 26 gauge clasp metal plate to the inlay or crown, setting it as far lingually and gingivally as possible—this strip to be 1/8 of an inch wide and 1/4 of an inch long. 1/16 of an inch from the end, bend this strip to an obtuse or nearly a right angle, after it is well annealed. This short part to set flat against the crown or inlay to insure a strong union when soldered—the long part to project parallel with the alveolar ridge and stand with the edge occluso-gingivally. The button to be soldered to the side of this strip, near the end, to project buccally or labially from the strip. By this method the button is placed near the gum and about 1/16 of an inch from the anchor tooth.

The contact spur on the tube must be cut off when the attachment is used in this manner and a new contact spur either of plate or wire soldered to the tube, following the technic described for the older form.

This is necessary because the contact spur provided on the attachment will not act as a contact spur because the tube is set edgewise or almost at a right angle with the proximal surface of the tooth, therefore a new contact spur must be provided.

Where two or more attachments are used they should of course be set as nearly parallel as possible, but no jig or paralleling device is necessary as absolute parallelism is not needed.

The oval shaped button of the new form, or the speroidal shaped button of the older form allows the tube sufficient movement under masticating stress to relieve the anchor tooth or teeth of undue strain.

Before closing on this subject I desire to state that I do not consider this the best form of attachment for all cases. Like practically every other good thing it has its limitations and is best suited for the class of cases I have described, viz., where all the molars and some of the bicuspids are missing, or anywhere where the end of the saddle or plate opposite from where attachment is made must be free, because of no natural tooth present to which attachment can be made. Where both ends of the appliance are supported by natural teeth the Roach attachment can be used, but has no special advantage over many other forms with the exception that it allows the artificial denture to settle without undue strain upon the anchor teeth, and that it is in my opinion one of the simplest and strongest of the patented retentive appliances. When it is used where both ends are supported with natural teeth in position, the contact spurs are not necessary. The plain tubes and buttons are sufficient. In such cases the buttons should not be soldered close to the gum line, but rather be set as near the occlusal portion of the teeth and as far lingually as the case will allow without interfering with the tongue. Where the artificial tooth next to the attachment is anterior enough to be conspicuous and there is not room enough to place a vulcanite tooth, a Steele facing can be used to advantage by soldering the backing of the Steele tooth to the metal saddle or to the anchor lug of the tube of the attachment and cementing the facing to position after the case is otherwise finished.

Considering now that class of cases where we wish to avoid mutilation of the tooth—where the tooth to which anchorage must be made is perfect. I will briefly describe a new form of clasp also devised by Dr. F. E. Roach, which he terms the mesio-distal grip clasp. As its name implies, it grips the tooth mesio-distally.

For cuspids and incisors it can be made reasonably effective with comparatively little show of metal. It can also be made to operate well on bicuspids and molars. For illustration I will take a cuspid because that type of tooth is usually difficult to clasp to good advantage. (The same general technic, however, is used for making these clasps for the posterior teeth.)

The first requisite is to obtain a good impression of the tooth to be clasped, either in plaster of paris or modeling compound. If the tooth is isolated, a plaster impression, using the technic described by Dr. Cummer, of Toronto, is an excellent method. If modeling compound is used the impression must be taken by the sectional or core method.

The cast should be made of some hard material; Melotte's or other fusible metal can be poured into a plaster impression. Copper amalgam, cement, Price's or Weinstein's artificial stone are excellent for making casts in modeling compound impressions. Thus a hard model upon which the clasp can be formed is obtained.

Having a true reproduction of the tooth, place on the lingual surface a piece of pure gold or platinum 36 to 40 gauge and burnish it to position—this to cover about 1/3 of the lingual surface. Now take a round clasp metal or elastic wire 16 to 17 gauge, bend it to a narrow U or hair pin form; place it on the mesial side of the tooth so the bent angle will project incisally of the contact point and the two bars extend gingivally, lying in the labial and lingual embrasures. Bend the lingual bar near its end to an obtuse or nearly right angle and slightly flatten so it will lie on the burnished piece of gold or platinum. Then tack it with hard sticky wax. Now remove it from the model, invest and unite with 18 karat solder, flowing sufficient solder to stiffen and strengthen the lingual portion. Then replace on the tooth model and fit another U shaped clasp metal or elastic wire 16 or 17 gauge on the distal, similar to that on the mesial; place it in position and tack with hard sticky wax. Now form an anchorage lug by flattening the end of a 14 gauge half-round wire about ½ an inch long, and bend it to lie properly over the burnished metal on the lingual of the tooth model, also bend wire so it will extend to the metal saddle to which it can be subsequently soldered, or so it will project into the vulcanite and be securely anchored. Now, slightly heat the flattened end so it will melt into position in the wax on the lingual plate of metal which forms part of the clasp; reinforce well with sticky wax, remove from tooth model, invest and solder, flowing sufficient solder to make a strong rigid union. The two loose bars of the clasp which lay in the mesial and distal labial embrasures are left comparatively loose, not tightly fitted until the partial denture is completed and placed in the patient's mouth, at which time any slight bending or fitting to make the clasp grip well can be accomplished with wire bending pliers. In fact these labial bars should be left reasonably loose while making the clasp, as this facilitates the removal of the parts while constructing the clasp, and also the denture when fitting it to place in the patient's mouth. (Where bicuspids or molars are clasped, the same general plan of construction is followed.) An expert with pliers can make this clasp out of one piece of wire to which is soldered an anchorage lug, but for the average man the technic I described above is much easier and the results usually better.

On the posterior teeth it is often advisable to solder one or two occlusal rests so the clasp will stay in its proper position on the tooth which is clasped. An expert may avoid the longer technic of making an accurate model of the tooth by fitting the clasp while constructing it directly on the patient's tooth, but this is not easy especially on the posterior teeth. Frequently it is necessary to cut a slight notch in the mesio or disto-incisal or occlusal angles of the tooth which is clasped or the approximating tooth or else slightly shorten the cusp or cusps of the antagonizing tooth or teeth to allow the wires which hook from the lingual embrasures to the labial or buccal sufficient room to prevent their interference with occlusion.

Sometimes too, after the case is completed we find that we can dispense with a portion of a labial or buccal bar because the artificial tooth on the denture makes tight contact with the tooth clasped, and the bar in the other embrasure will grip sufficient to retain the appliance. Where this can be done, it is advisable to do so for it thereby prevents the show of extra metal. The end of the bar which lies in the labial or buccal embrasure can, if necessity requires, be modified by soldering to it a narrow strip of 26 gauge clasp plate or 17 gauge wire, so shaped as to fit the gingival part of the clasped tooth, or of the approximating tooth. This will sometimes give extra retention and stability to the appliance.

There is of course this objection to be made to this form of clasp, viz., that it covers considerable of the lingual portion of the enamel, and that it forms a rather rigid attachment to the tooth clasped, thereby subjecting the tooth to overstrain if there is much leverage produced by a large plate or a saddle which is long and unsupported at the other end.

All clasps are more or less objectionable in some manner or other, but for an attachment to a healthy natural tooth in a mouth reasonably immune to caries and where the patient will remove the bridge or plate every night and apply prophylactic measures, it is the best form of clasp attachment known to the writer, especially for the anterior teeth, those which have given us most trouble to clasp well.

These simple, yet effective, retentive appliances allow the greatest possible conservation of tooth structure and vital pulps—the latter especially being a most important factor.

